# Workplace AI Usage, Intrinsic Motivation, and Job Burnout: Evidence from a Longitudinal Study

**DOI:** 10.3390/bs16091587

**Published:** 2026-09-07

**Authors:** Kaiyue Wang, Yongxin Li

**Affiliations:** 1 Faculty of Health and Wellness, City University of Macau, Macau 999078, China; h24092100152@cityu.edu.mo; 2 Institute of Psychology and Behavior, Henan University, Kaifeng 475004, China

**Keywords:** artificial intelligence usage, intrinsic motivation, job burnout, conservation of resources theory

## Abstract

Background: With the widespread application of artificial intelligence technology in organizational contexts, understanding its implications for employees’ motivation and well-being has become increasingly important. Yet, how employees’ AI usage is prospectively associated with intrinsic motivation and job burnout remains insufficiently understood. Based on conservation of resources theory, this study adopted a two-wave longitudinal design to examine the relationships among employees’ artificial intelligence usage, intrinsic motivation, and job burnout. Methods: Data were collected through a two-wave questionnaire survey. The final sample consisted of 313 employees. A cross-lagged panel model was adopted for analysis. Results: AI usage positively predicted subsequent job burnout and negatively predicted subsequent intrinsic motivation after accounting for prior levels and other model variables. Intrinsic motivation negatively predicted subsequent job burnout. In addition, higher job burnout positively predicted subsequent AI usage, indicating a potential bidirectional prospective relationship between AI usage and job burnout. The sensitivity analysis showed a generally similar directional pattern, although the job burnout to AI usage pathway was attenuated and became nonsignificant. Conclusions: Artificial intelligence usage should not be viewed solely as a job resource. Although it may enhance work efficiency and convenience, it may also be associated with higher subsequent job burnout and lower intrinsic motivation. Intrinsic motivation may represent an important psychological pathway linking AI usage to later job burnout.

## 1. Introduction

In recent years, the application of artificial intelligence (AI) in the workplace has become increasingly widespread and has emerged as an important strategy for organizations to enhance productivity and value creation (Bankins et al., 2024). Recent survey data show that about 88% of surveyed organizations regularly used AI in at least one business function in 2025, representing an increase from 78% in the previous year (McKinsey & Company, 2025). With the emergence of intuitive and user-friendly conversational AI tools such as ChatGPT, AI usage has grown rapidly in organizations and has become increasingly embedded in professional fields such as education (Hashmi & Bal, 2024), tourism (Gursoy et al., 2023), and innovation management (Sedkaoui & Benaichouba, 2026). Therefore, AI has become an important working tool for modern organizations, providing new opportunities for employees and organizations to improve efficiency, support decision-making and create value. AI technologies are described as a transformative force that can accelerate innovation (Li et al., 2026), improve productivity and enhance quality of life in multiple fields (Sedkaoui & Benaichouba, 2026). Dwivedi et al. (2023) suggest that AI can play an important role in brainstorming by analyzing large volumes of information and generating summaries from diverse perspectives. Thus, AI can provide valuable support for employees by improving efficiency and expanding their problem-solving capacity, while also reshaping the way employees experience and engage in their work (Wu et al., 2025).

However, alongside the benefits of AI adoption, its potential psychological costs have begun to attract scholarly and practical attention. Y. Liu et al. (2024) found that AI adoption may simultaneously increase employees’ workplace anxiety and enhance their technological self-efficacy, which leads to both proactive service behavior and work withdrawal behavior. Similarly, Shin et al. (2025) suggested that the use of AI in management operations may make employees feel that their humanity is denied and they are treated merely as numbers, thereby increasing the sense of organizational dehumanization and triggering negative reactions. Although AI may provide employees with unprecedented work convenience, technological change is also reshaping their work content, requiring employees to adjust their work routines and continuously invest in learning, monitoring and adaptation. These demands create feelings of threat, tension, and uncertainty (Wu et al., 2024). As a result, there may emerge a paradoxical pattern: employees may use AI to improve efficiency, while greater AI usage may also be accompanied by higher levels of adaptation pressure and psychological strain (Zheng & Zhang, 2025). This paradox is consistent with the evidence that AI technostress can increase exhaustion, intensify work-family conflict and reduce job satisfaction, even if AI usage continues to help improve productivity (Chuang et al., 2025).

Conservation of Resources (COR) theory provides a framework for explaining this process. Research shows that technological iteration and organizational change may intensify employees’ existing workloads, impose new demands on their skill systems, and negatively influence employees’ emotions, well-being, and burnout levels (Tarafdar et al., 2007). According to COR theory, individuals will strive to acquire, maintain, and protect valuable resources. When the work context continuously consumes employees’ cognitive, emotional and motivational resources, employees may experience stress responses and job burnout. Job burnout is a chronic work-related psychological state characterized by emotional exhaustion, cynicism, and reduced professional efficacy (Bottini et al., 2025). It is an important indicator of employee well-being because it reflects sustained resource depletion and may impair employees’ motivation, work engagement, and long-term job performance (González-Romá et al., 2006). Therefore, examining how AI usage is related to job burnout is important for understanding the psychological implications of AI-enabled work and for helping organizations promote more sustainable human–AI collaboration.

Although the existing literature has already acknowledged the double-edged effects of AI (Duan et al., 2026b), several issues remain insufficiently clarified. First, prior studies have linked workplace AI usage to employees’ job burnout, but many of them have relied on cross-sectional designs. For example, H. Liu et al. (2024) found in a cross-sectional study that frequent AI usage was associated with a higher risk of job burnout among radiologists. Similarly, Gu et al. (2026) reported, based on a cross-sectional study of university students, that AI technology dependence was positively associated with learning burnout. Such designs can identify associations between AI usage and job burnout, but they provide limited evidence regarding temporal ordering. It remains unclear whether AI usage predicts employees’ later job burnout after accounting for prior job burnout levels. This limitation is important because AI usage may not only reflect employees’ current work conditions but may also be associated with their subsequent psychological states. By adopting a two-wave cross-lagged design, this study examines the prospective association between AI usage and job burnout while controlling for their prior levels.

Second, existing research has explained the negative consequences of AI usage from the perspectives of external work-related pressures, such as technostress, job insecurity, increased job demands, or human–AI collaboration challenges (Brougham & Haar, 2018). However, less attention has been paid to how AI usage affects employees’ internal motivational resources. Self-determination theory (SDT) provides an explanation for how AI usage may shape intrinsic motivation by influencing employees’ experiences of autonomy and competence at work. When employees use AI extensively to complete work tasks, they may experience reduced autonomy, weakened task involvement, or a lower sense of personal contribution (Gagné et al., 2022). In this process, work may become less intrinsically meaningful, and employees may gradually lose interest and psychological involvement in their tasks. From a COR perspective, intrinsic motivation can be regarded as a motivational resource because it reflects employees’ interest, enjoyment, and autonomous engagement in their work (Grant, 2008). It facilitates employees’ goal-directed work behavior and contributes to work-related well-being (Halbesleben et al., 2014; Stockkamp & Kuonath, 2023). A decline in intrinsic motivation may therefore reflect a reduction in employees’ motivational resources, leaving fewer psychological resources available to sustain their engagement with work and increasing their susceptibility to job burnout. Relative research has shown that job burnout was dynamically associated with reduced basic psychological need satisfaction and meaning in work, highlighting the role of internal motivational resources in job burnout development (Maunz & Glaser, 2024). However, it remains unclear whether this motivational resource process also applies to AI-enabled work, particularly whether AI usage may be associated with job burnout through lower intrinsic motivation. Other research has examined how AI awareness affects job burnout and found that perceived organizational support and organizational commitment mediate this relationship (Wang & Zhou, 2025). Such findings primarily explain job burnout through resources and external pressures at the organizational level, leaving it unclear how AI-related experiences may deplete employees’ internal motivational resources. To address this gap, this study examines whether intrinsic motivation contributes to a prospective indirect association between AI usage and subsequent job burnout. By examining the indirect role of intrinsic motivation, this study suggests that the prospective association between AI usage and subsequent job burnout may involve lower intrinsic motivation, in addition to the external pressures emphasized in prior research.

Third, prior studies mainly examined how collaboration with AI influences employees’ psychological experiences, such as stress, well-being, and job burnout (Cajnko & Cajnko, 2025; Sharif et al., 2025), but rarely considered whether these psychological states may in turn associate with employees’ subsequent AI usage. This one-directional perspective may be insufficient because employees’ use of AI is not only a response to technological availability or organizational demands but may also reflect their current states of psychological resources. From the COR perspective, job burnout represents a state of sustained resource depletion. Employees who have experienced job burnout often have limited emotional energy, reduced initiative, and weakened motivation to make effort in work tasks (Bakker et al., 2014). Under such conditions, they may become more likely to rely on AI to reduce effort, simplify work processes, or compensate for depleted personal resources. Therefore, it is important to examine whether job burnout predicts later AI usage and clarify whether such a prospective association may be consistent with compensatory or avoidance-oriented responses to resource depletion, rather than viewing AI usage solely as an antecedent of employee well-being. This perspective further highlights the potential interplay between resource depletion and employees’ responses to resource loss in human–AI collaboration. It also provides practical insights for organizations to preserve employees’ psychological resources while promoting AI adoption.

## 2. Theoretical Review and Research Hypotheses

### 2.1. Employee AI Usage and Job Burnout

COR theory posits that individuals have the fundamental motivation to acquire, retain, and protect valued resources (Hobfoll, 1989). According to this theory, individuals will feel stressed when they perceive the threat of resource loss, experience actual resource loss or fail to obtain adequate returns on their resource investments. Moreover, this stress could negatively affect their work outcomes and well-being. In current digital workplaces, AI usage can be regarded as a process of resource investment. By leveraging AI, employees can gain access to useful resources, such as information processing, knowledge integration, task automation and decision support, which can help save time and cognitive effort (Duan et al., 2026a; Zhang et al., 2026). However, AI usage is not only a source of resource acquisition. This dual role of AI usage is also consistent with the Job Demands–Resources model, which suggests that workplace characteristics can function as job resources that facilitate goal attainment or as job demands that require sustained effort and may incur psychological costs (Bakker & Demerouti, 2017). From this perspective, AI may provide instrumental resources while simultaneously introducing additional cognitive, learning, and adaptation demands. As AI becomes increasingly embedded in organizational work, it creates new demands that may threaten the valuable resources of employees. The rapid advancement of AI, robotics and algorithmic technologies can heighten employees’ concerns about job replacement and career insecurity (Brougham & Haar, 2018). Meanwhile, the integration of AI into daily work requires employees to constantly acquire new technological competencies, adapt to evolving work processes, and collaborate effectively with intelligent systems. All of these require sustained investments of cognitive and learning resources (Lin & He, 2024). Since job security, professional competence, career prospects and perceived control are valuable personal resources, threats to these resources may trigger psychological strain and defensive responses (Hobfoll, 1989). For example, research demonstrates that AI-induced job insecurity could reduce the sense of psychological safety while increasing knowledge hiding behavior and undermining employees’ well-being and career outcomes (Kim & Kim, 2024; Sharif et al., 2025; Wang & Zhou, 2025). Rather than serving solely as a resource that enhances work efficiency, AI usage also requires employees to make continuous resource investments, which exposes them to the possibility of resource loss. From a COR perspective, when these resource demands exceed employees’ capacity to replenish depleted resources, AI usage is likely to accelerate resource depletion.

One unignorable consequence of prolonged resource depletion is job burnout. Job burnout is a psychological response to continuous work stress, characterized by emotional exhaustion, cynicism and reduced professional efficacy (Schutte et al., 2000). Greater involvement with AI at work may be accompanied by greater resource demands, including technology learning, performance comparison, job replacement concerns, and continuous adjustments to work practices. When employees are unable to derive sufficient resources (i.e., competence, control, or organizational support) from AI usage, it may shift from functioning as a job resource to a job demand, thereby intensifying psychological strain. Consistent with this point, AI-related psychological experiences, such as AI awareness and AI anxiety, have been shown to be associated with emotional exhaustion, depression, and burnout (Chen et al., 2025; Teng et al., 2024; Zheng & Zhang, 2025). Taken together, greater AI usage may place greater demands on employees’ resources and is therefore expected to predict higher subsequent job burnout. Therefore, we propose the following hypothesis:

**H1.**  *Employee AI usage positively predicts subsequent job burnout.*

### 2.2. The Mediating Role of Intrinsic Motivation

Intrinsic motivation can be regarded as an important psychological resource because it reflects employees’ internal interest, sense of meaning, and willingness to invest effort in their work. Complementing the COR perspective, SDT provides an additional explanation for the association between AI usage and intrinsic motivation (Gagné & Deci, 2005). As a key driver of work engagement and persistence, intrinsic motivation develops when employees experience a sense of autonomy and competence in their work (Gagné et al., 2022). When these basic psychological needs are fulfilled, employees are more likely to remain interested, enthusiastic, and fully engaged in their work. In contrast, when autonomy and competence are undermined, work is more likely to be perceived as an external demand rather than a personally meaningful activity, resulting in greater psychological strain and an increased risk of burnout (Rubino et al., 2009). Consistent with this view, satisfaction of employees’ needs for autonomy, competence, and meaningful work has been shown to be negatively associated with job burnout, whereas higher levels of job burnout may further erode the fulfillment of these psychological needs and employees’ sense of work meaning (Maunz et al., 2024). Moreover, workplace factors can influence emotional exhaustion by shaping employees’ basic psychological need satisfaction and intrinsic motivation (Maunz & Glaser, 2024).

AI usage may reduce employees’ intrinsic motivation through several pathways. At the organizational level, AI adoption has been shown to weaken employees’ relational psychological contracts and trust in organizations, thereby reducing work engagement (Braganza et al., 2021). More importantly, the widespread integration of AI into work requires employees to continuously adapt to new technologies, workflows, and performance standards. When employees perceive that AI constrains their autonomy, substitutes for their professional expertise, or diminishes the meaningfulness of their work, they are less likely to engage in work out of genuine interest and self-determination, leading to lower intrinsic motivation (Gagné et al., 2022). AI usage may therefore frustrate employees’ needs for autonomy and competence, thereby reducing intrinsic motivation. Lower intrinsic motivation, in turn, limits employees’ willingness to invest energy in their work and leaves them more vulnerable to job burnout. From the perspective of COR theory, this process reflects a loss of motivational resources. Taken together, SDT provides a complementary account of how AI usage may undermine intrinsic motivation, while COR theory explains why such a reduction in motivational resources may be associated with greater subsequent job burnout. Accordingly, AI usage is expected to negatively predict employees’ subsequent intrinsic motivation, and intrinsic motivation is expected to contribute to a prospective indirect association between AI usage and subsequent job burnout.

**H2.**  *Intrinsic motivation contributes to a prospective indirect association between employee AI usage and subsequent job burnout.*

### 2.3. Job Burnout and Employee AI Usage

Job burnout not only reflects a state of emotional exhaustion and psychological detachment from work but also has important implications for employees’ subsequent work behaviors. Longitudinal evidence suggests that job burnout can undermine subsequent job performance and functioning (Bakker et al., 2014). Maunz et al. (2026) using five-wave longitudinal data found that employees experiencing higher levels of job burnout became less proactive and less effective in accomplishing work tasks, making them more likely to perceive or encounter additional job demands, including workload, social stressors, and organizational stressors. Employees experiencing job burnout often have diminished energy and reduced willingness to sustain effort at work (Gagné et al., 2022). Under such circumstances, relying solely on their own cognitive and emotional resources to complete work tasks becomes increasingly costly. From a COR perspective, employees therefore tend to reduce the effort required to accomplish work goals and seek external resources that can compensate for depleted personal resources. As AI becomes increasingly integrated into daily work routines, it provides employees with accessible support for information processing, content generation, and task completion (Chuang et al., 2025). Consequently, employees with higher levels of job burnout may increase their subsequent AI usage as a compensatory resource to cope with resource depletion while alleviating workload and maintaining work performance. It may also partly reflect an avoidance-oriented response, as employees with lower energy and motivation may prefer work strategies that reduce the effort required for cognitively demanding tasks. Accordingly, higher levels of job burnout are expected to positively predict subsequent AI usage.

**H3.**  *Employee job burnout positively predicts subsequent AI usage.*

As AI technologies become increasingly prevalent in organizational environments, employees’ interactions with AI have emerged as an important factor shaping their work experiences and psychological states. Although existing research has acknowledged the double-edged effects of AI, several issues remain insufficiently understood. Specifically, how AI usage is prospectively related to employees’ internal motivational resources and whether employees’ psychological states may in turn predict their subsequent AI usage remains unclear. To address these gaps, this study develops a cross-lagged model incorporating AI usage, intrinsic motivation, and job burnout to examine the prospective relationships among these constructs and the potential bidirectional prospective associations between AI usage and job burnout, primarily based on COR theory and complemented by SDT.

## 3. Methods

### 3.1. Data Collection

This study employed a convenience sampling method and recruited enterprise employees who had exposure to and experience using artificial intelligence in their work. Participants were recruited from organizations located in Chongqing, Zhejiang, Guangdong, and Henan, China, and covered a range of job positions, including managerial, operational, marketing, financial and professional roles. Before filling out the questionnaire, participants were required to read an informed consent statement on the first page of the survey. The statement explained the purpose of the study, the voluntary nature of participation, the anonymity and confidentiality of responses, and that the data collected would be used for academic research purposes only. Participants were able to proceed to the formal questionnaire only after indicating their informed consent. A two-wave longitudinal design was adopted. The first wave of data collection was conducted among employees who met the inclusion criterion of having work-related AI usage experience. One month later, the second wave of data collection was carried out using the same procedure. A one-month interval was selected to capture relatively proximal prospective associations in employees’ motivational resources and job burnout. Similar time intervals have been used in previous longitudinal research examining resource processes and job burnout (Otto et al., 2021), and are consistent with recommendations that longitudinal time lags should reflect the temporal characteristics of the processes under study (Dormann & Griffin, 2015; Mitchell & James, 2001). To match participants’ responses across the two waves while protecting their privacy, respondents were asked to provide the last four digits of their mobile phone numbers. This information was only used for data matching and not used to identify individual participants. After data matching and screening, responses from participants who completed both waves were retained for subsequent analysis. To encourage participation, a small monetary incentive was provided after questionnaire completion.

A total of 401 questionnaires were collected in the first wave. Second-wave questionnaires were distributed to the same participants in the first survey, and 385 questionnaires were collected in the second wave. After matching and data screening, 313 valid responses were retained for analysis. The follow-up response rate was 96.0%, and the valid response rate was 81.3%. In the final sample, 42.5% of participants were male, and 57.5% were female. The majority of participants were between 26 and 40 years old, accounting for 63.7% of the sample. Most participants held a bachelor’s degree (60.6%), while 21.2% held a master’s degree or above.

### 3.2. Measures

All measures were assessed using a seven-point Likert scale ranging from 1 (“strongly disagree”) to 7 (“strongly agree”).

AI usage was measured using the three-item AI usage scale developed by Tang et al. (2022). Consistent with the original conceptualization, AI usage refers to the extent to which employees use and spend time with AI in the pursuit of work goals. Thus, the scale is intended to capture employees’ overall degree of involvement with AI in their work activities, including the extent to which AI is incorporated into job functions, time spent working with AI, and involvement of AI in important work decisions. Items include “I use AI to accomplish most of my job functions”, “I spent most of the time working with artificial intelligence”, “I worked with artificial intelligence in making major work decisions”. In this study, the Cronbach’s alpha coefficient for this scale was 0.870 at Time 1 and 0.899 at Time 2.

Intrinsic motivation was measured using the four-item work intrinsic motivation scale developed by Grant (2008). This scale assesses employees’ internal motivation to engage in work based on interest, enjoyment, and a sense of engagement. Sample items include “Because I enjoy the work itself” and “Because I find the work interesting”. Higher scores indicate higher levels of work intrinsic motivation. In this study, the Cronbach’s alpha coefficient for this scale was 0.948 at Time 1 and 0.947 at Time 2.

Job burnout was measured using the Maslach Burnout Inventory-General Survey (MBI-GS) developed by Schutte et al. (2000). The scale is suitable for general occupational populations and consists of 15 items across three dimensions: emotional exhaustion, cynicism, and reduced professional efficacy. Higher scores indicate higher levels of job burnout. Emotional exhaustion and cynicism were scored using their corresponding items, reduced professional efficacy was reverse-scored, and an overall observed burnout composite score was used in the cross-lagged analyses. Given the established multidimensional structure of burnout, the three dimensions were retained separately in the measurement analyses, whereas the overall composite score was used in the cross-lagged analyses to represent overall job burnout, consistent with previous longitudinal research employing an overall burnout representation (Kalimo et al., 2003; Wekenborg et al., 2019). Sample items include “I feel emotionally drained from my work.” In this study, Cronbach’s alpha coefficients were 0.923 at Time 1 and 0.949 at Time 2.

## 4. Data Analysis and Results

### 4.1. Common Method Bias Test

As the main variables were assessed through self-report measures, several steps were taken to minimize the potential influence of common method bias. During data collection, participants were informed that their responses would remain anonymous, which helped reduce concerns about the assessment. To further limit systematic response tendencies, the questionnaire was designed carefully, with some items reverse-scored, and the presentation order of the items was randomized to make the research purpose less obvious to participants. Harman’s single-factor test was conducted as a preliminary assessment to assess potential common method bias using SPSS 27. An unrotated exploratory factor analysis of the three key variables: AI usage, intrinsic motivation, and job burnout, extracted four factors with eigenvalues greater than 1. The first factor accounted for 38.99% of the total variance, which was below the commonly used threshold of 40%. As no single factor accounted for the majority of the variance, the results provided preliminary evidence against a substantial single-factor common method effect.

### 4.2. Descriptive Statistics and Correlation Analysis

Table 1 presents the means, standard deviations and correlation coefficients for AI usage, intrinsic motivation and job burnout across the two measurement waves. The results showed that T1 AI usage was significantly positively correlated with T2 AI usage, T1 intrinsic motivation was significantly positively correlated with T2 intrinsic motivation, and T1 job burnout was significantly positively correlated with T2 job burnout, indicating satisfactory temporal stability of the three constructs. In addition, AI usage was positively correlated with job burnout, whereas intrinsic motivation was negatively correlated with job burnout. The higher mean level of burnout at Time 2 may reflect short-term temporal variation, as burnout has been shown to fluctuate over time in response to changing work conditions (Hu et al., 2017; Schaufeli et al., 2009; Teuchmann et al., 1999).

### 4.3. Confirmatory Factor Analysis and Discriminant Validity

Confirmatory factor analyses were conducted separately at each measurement occasion to examine the factorial distinctiveness of AI usage, intrinsic motivation, and the three dimensions of job burnout. Given the theoretically established multidimensional structure of burnout (Maslach et al., 2001), exhaustion, cynicism, and reduced professional efficacy were modeled as separate first-order factors alongside AI usage and intrinsic motivation. The hypothesized five-factor model showed satisfactory fit at both Time 1, χ^2^(199) = 422.845, CFI = 0.951, TLI = 0.944, RMSEA = 0.060, SRMR = 0.042, and Time 2, χ^2^(199) = 400.087, CFI = 0.959, TLI = 0.952, RMSEA = 0.057, SRMR = 0.036 (see Table 2). All standardized factor loadings were statistically significant, ranging from 0.677 to 0.954 at Time 1 and from 0.736 to 0.983 at Time 2. Among the burnout dimensions, reduced professional efficacy showed the strongest latent correlation with intrinsic motivation at both Time 1 (r = −0.613) and Time 2 (r = −0.675), warranting a more stringent test of their discriminant validity. Accordingly, an alternative four-factor model was estimated in which intrinsic motivation and reduced professional efficacy were specified as a single factor. This alternative model showed substantially poorer fit at both Time 1, χ^2^(203) = 1071.174, CFI = 0.812, TLI = 0.786, RMSEA = 0.117, SRMR = 0.093, and Time 2, χ^2^(203) = 917.764, CFI = 0.853, TLI = 0.832, RMSEA = 0.106, SRMR = 0.069. These model comparisons support the empirical distinctiveness of intrinsic motivation from the job burnout dimensions. Additional discriminant-validity statistics provided consistent evidence. AVE values ranged from 0.672 to 0.884, composite reliability values ranged from 0.879 to 0.974, and HTMT values between intrinsic motivation and the three job burnout dimensions ranged from 0.387 to 0.687 across the two waves, all below the conservative 0.85 threshold.

HTMT values between AI usage and intrinsic motivation ranged from 0.095 to 0.132 across the two waves; those between AI usage and the three burnout dimensions ranged from 0.060 to 0.388; and those between intrinsic motivation and the burnout dimensions ranged from 0.387 to 0.687. All values were below the conservative 0.85 threshold, providing evidence of discriminant validity among the focal constructs.

### 4.4. Measurement Invariance

Measurement invariance was examined for AI usage, job burnout, and intrinsic motivation across the two measurement waves using Mplus 8.3. Given the multidimensional nature of job burnout, emotional exhaustion, cynicism, and reduced professional efficacy were modeled as separate latent factors at each wave. All latent constructs were measured using their corresponding scale items as observed indicators. As shown in Table 3, the measurement models of the three focal constructs achieved configural, metric, and scalar invariance across the two waves (ΔCFI ≤ 0.010, ΔRMSEA ≤ 0.015, ΔSRMR ≤ 0.015). These findings provide qualified support for longitudinal measurement invariance across the two measurement occasions.

### 4.5. Model Test

The primary cross-lagged panel model (CLPM) was estimated using observed composite scores for AI usage, intrinsic motivation, and overall job burnout. Age and income were included as covariates predicting all three Time 2 variables. All autoregressive and cross-lagged paths, covariances among the Time 1 variables and covariates, and Time 2 residual covariances were freely estimated. The model was therefore saturated.

As shown in Table 4, after controlling for the autoregressive effects of each variable as well as age and income, T1 AI usage significantly and positively predicted T2 job burnout (β = 0.110, *p* = 0.002) and significantly and negatively predicted T2 intrinsic motivation (β = −0.111, *p* = 0.019). In addition, T1 intrinsic motivation significantly and negatively predicted T2 job burnout (β = −0.177, *p* < 0.001). Furthermore, T1 job burnout significantly and positively predicted T2 AI usage (β = 0.142, *p* = 0.011). Among the non-hypothesized cross-lagged paths, T1 job burnout negatively predicted T2 intrinsic motivation (β = −0.119, *p* = 0.038), whereas T1 intrinsic motivation did not significantly predict T2 AI usage (β = 0.038, *p* = 0.464). These findings indicate prospective direct and indirect associations between AI usage and subsequent job burnout, with intrinsic motivation contributing to the indirect pathway. Meanwhile, higher levels of job burnout predicted subsequent AI usage, indicating bidirectional prospective associations between AI usage and job burnout in the primary model. Neither age nor income significantly predicted any of the endogenous variables. Although the zero-order correlation between Time 1 AI usage and Time 2 intrinsic motivation was negative but nonsignificant (r = −0.066), Time 1 AI usage significantly predicted lower Time 2 intrinsic motivation after accounting for prior intrinsic motivation and other predictors (β = −0.111, *p* = 0.019). This pattern is consistent with statistical suppression, suggesting that the negative prospective association became more pronounced after accounting for the other variables in the model. To evaluate the incremental contribution of AI usage to subsequent job burnout beyond the autoregressive effect and other predictors, the full model was compared with a restricted model in which the T1 AI usage to T2 job burnout path was omitted. The full model explained approximately 74.5% of the variance in T2 job burnout, compared with approximately 73.6% in the restricted model, corresponding to an incremental ΔR^2^ of approximately 0.010. Thus, T1 AI usage accounted for approximately 1% additional variance in subsequent job burnout beyond prior job burnout, intrinsic motivation, age, and income.

**Figure 1:**
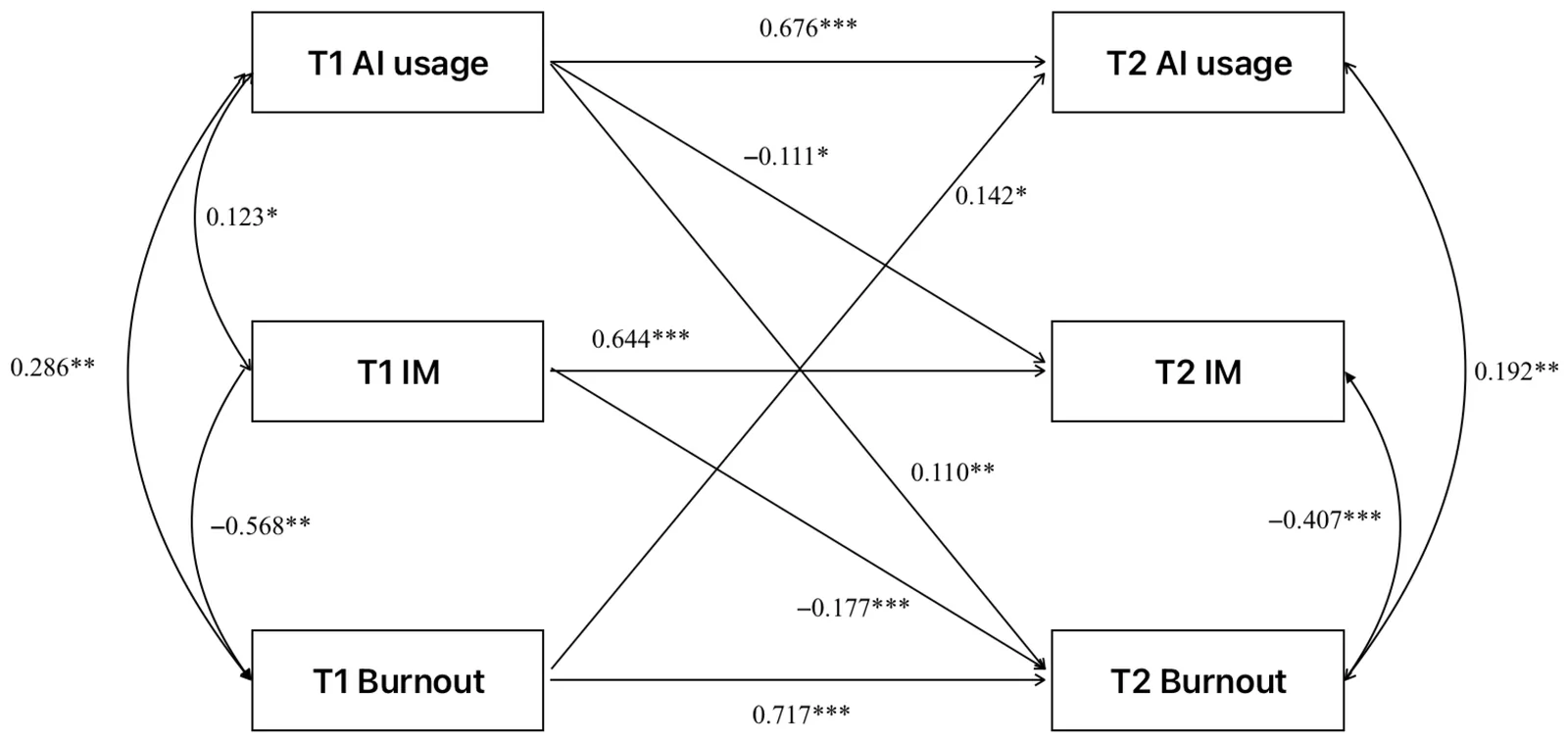
Cross-Lagged Panel Model. Note: Only the focal hypothesized cross-lagged paths are displayed for clarity. All cross-lagged paths were estimated in the model. * *p* < 0.05, ** *p* < 0.01, *** *p* < 0.001 (two-tailed).

To further examine the half-longitudinal mediating role of intrinsic motivation, a bootstrap analysis with 5000 resamples was conducted (see Figure 1). Following Cole and Maxwell’s (2003) two-wave half-longitudinal approach, the prospective indirect association was estimated as the product of the path from Time 1 AI usage to Time 2 intrinsic motivation and the path from Time 1 intrinsic motivation to Time 2 job burnout, with the prior level of each outcome controlled, under the assumption that the corresponding lagged relations remain stable across adjacent time intervals. The results indicated that the indirect effect of AI usage on job burnout through intrinsic motivation was significant (b = 0.074, 95% CI [0.007, 0.140]), as the confidence interval did not include zero. These findings indicate that AI usage was prospectively associated with subsequent job burnout through lower intrinsic motivation, supporting the half-longitudinal mediating role of intrinsic motivation in the relationship between AI usage and job burnout.

As a supplementary analysis, the three dimensions of job burnout were examined separately using the same cross-lagged model specification. T1 AI usage significantly predicted higher T2 cynicism (β = 0.124, *p* = 0.003) and higher T2 reduced professional efficacy (β = 0.303, *p* < 0.001), whereas its prospective association with T2 emotional exhaustion was weaker and did not reach statistical significance (β = 0.075, *p* = 0.076). These findings indicate some variation in the prospective associations between AI usage and different dimensions of burnout. These findings indicate that the prospective association between AI usage and job burnout was not uniform across the three dimensions and was more pronounced for reduced professional efficacy than for cynicism or emotional exhaustion.

### 4.6. Sensitivity Analysis

As a sensitivity analysis, a hybrid latent-observed CLPM was estimated in which AI usage and intrinsic motivation were modeled as latent variables across the two waves, with equality constraints imposed on factor loadings and item intercepts. Job burnout was retained as an observed composite because representing all job burnout items with a single latent factor would oversimplify its established multidimensional structure. In addition, reduced professional efficacy was less strongly associated with exhaustion and cynicism than these two dimensions were with each other, consistent with previous research suggesting a comparatively distinct role of professional efficacy within job burnout (Lee & Ashforth, 1996; Schaufeli & Salanova, 2007). Therefore, treating all burnout items as indicators of a single latent burnout factor was not considered appropriate. The observed composite was therefore used to represent overall burnout in the structural model without imposing a single-factor latent measurement structure, consistent with previous longitudinal research using an overall burnout score (Kalimo et al., 2003; Wekenborg et al., 2019). The hybrid model showed good overall fit, χ^2^(122) = 191.872, CFI = 0.986, TLI = 0.983, RMSEA = 0.043, and SRMR = 0.041. The main pattern of results was retained: T1 AI usage negatively predicted T2 intrinsic motivation (β = −0.130, *p* = 0.009) and positively predicted T2 job burnout (β = 0.116, *p* = 0.003), while T1 intrinsic motivation negatively predicted T2 job burnout (β = −0.196, *p* < 0.001). The half-longitudinal indirect association between T1 AI usage and T2 job burnout through intrinsic motivation was also significant (B = 0.305, *p* = 0.014, 95% bias-corrected bootstrap CI [0.103, 0.616]). In contrast, the path from T1 job burnout to T2 AI usage was positive but nonsignificant (β = 0.072, *p* = 0.073).

## 5. Discussion

This study showed that AI usage prospectively predicted slightly greater job burnout and was also indirectly associated with subsequent job burnout through lower intrinsic motivation. Specifically, greater AI usage predicted lower subsequent intrinsic motivation after accounting for prior intrinsic motivation and other model variables, while intrinsic motivation was negatively associated with subsequent job burnout. These findings suggest that intrinsic motivation, as an important psychological resource, may represent one mechanism through which AI usage is associated with later job burnout. The primary CLPM further showed that higher job burnout prospectively predicted greater subsequent AI usage, suggesting prospective associations in both directions between AI usage and job burnout. This reverse association was attenuated and became nonsignificant in the sensitivity analysis, indicating that the burnout-to-AI usage pathway was less robust across model specifications.

First, the present study identified prospective associations between AI usage and job burnout, with the primary model providing support for Hypotheses 1 and 3. Specifically, T1 AI usage positively predicted T2 job burnout, indicating a prospective association between greater AI usage and higher subsequent job burnout. This finding is consistent with previous research indicating that although AI technologies can improve work efficiency, they may also expose employees to greater technostress, concerns about job displacement, and resource depletion, thereby increasing the risk of psychological strain and job burnout (Brougham & Haar, 2018; He et al., 2025). One possible explanation is that greater AI usage may be accompanied by additional cognitive demands on employees. Rather than simply accepting AI outputs, employees are often required to evaluate, verify, revise and integrate AI-generated content into their work. When AI outputs are inaccurate or fail to meet task requirements, employees have to invest extra cognitive effort to review and revise the results (Klonek & Parker, 2025). These additional demands may help explain why greater AI usage was prospectively associated with higher levels of subsequent job burnout. Another possible explanation is that AI usage may heighten employees’ perceptions of career uncertainty. As AI becomes more and more involved in core work activities, employees may become more concerned that their skills and professional value are being challenged. Such perceived replacement threats may be associated with lower perceived control and job security, as well as greater tension and anxiety. Employees who perceive greater AI-related replacement threats may also experience higher job insecurity and career-related anxiety, which may be associated with greater emotional exhaustion and job burnout (Wang & Zhou, 2025; Zheng & Zhang, 2025). The psychological consequences of AI usage, however, may depend on whether AI functions primarily as a job resource or creates additional job demands. AI may reduce workload and facilitate task completion in some contexts, while in others it may introduce additional cognitive, coordination, or monitoring demands. Thus, the present finding should not be interpreted as indicating that AI usage is inherently detrimental. Rather, its association with employee burnout may depend on how AI is incorporated into work design and the extent to which its resource-providing benefits outweigh the demands associated with its use.

The dimension-level findings further qualify the prospective association between AI usage and overall job burnout. It was most pronounced for reduced professional efficacy, weaker but statistically significant for cynicism, and nonsignificant for emotional exhaustion. Taken together with the negative prospective association between AI usage and intrinsic motivation, this pattern suggests that AI usage may be more closely associated with employees’ motivation and competence-related self-evaluations than with emotional exhaustion over this interval. Reduced professional efficacy reflects negative evaluations of one’s competence and accomplishment at work (Maslach et al., 2001), whereas SDT identifies feelings of competence as important for sustaining intrinsic motivation (Gagné & Deci, 2005). As digital technologies can reshape opportunities for skill use and influence employees’ need satisfaction and motivation (Gagné et al., 2022), one possible interpretation is that, when employees perceive AI as limiting opportunities to exercise their abilities or make a distinctive personal contribution, they may experience both lower intrinsic motivation and a weaker sense of professional efficacy. The positive association with cynicism may reflect psychological distancing from work, which is a defining feature of the cynicism dimension of burnout (Maslach et al., 2001). Related evidence comes from a recent study using the MBI-GS cynicism subscale, which found that AI-supported work was indirectly associated with greater cynicism through hindrance appraisal and job replacement anxiety (Narayanan et al., 2026). By contrast, the nonsignificant association with emotional exhaustion suggests that greater AI usage was not uniformly associated with all aspects of burnout over this interval. One possible explanation is that emotional exhaustion reflects accumulated emotional depletion (Maslach et al., 2001), and its prospective association with AI usage may therefore require a longer observation period to emerge.

The primary CLPM showed that T1 job burnout positively predicted T2 AI usage, suggesting that employees with higher levels of job burnout may be more likely to use AI in subsequent work. Although this pathway was not consistently supported across alternative model specifications, its direction in the primary model raises several possible explanations. According to COR theory, individuals experiencing resource loss or depletion tend to adopt strategies to conserve their remaining resources and minimize further losses. For employees with depleted energy and reduced work engagement, AI can function as an external instrumental resource that helps alleviate task demands, save time and maintain basic work performance. Meanwhile, job burnout may reduce employees’ willingness to invest effort in cognitively demanding tasks, leading them to prefer work approaches that require less effort or provide greater technological assistance. Employees experiencing job burnout often lack the energy and motivation needed for sustained problem-solving, creative thinking and complex information processing. In such situations, AI can provide rapid feedback and preliminary solutions, thereby lowering the effort required to initiate and complete work tasks. Therefore, employees with higher levels of job burnout may increasingly rely on AI to cope with the imbalance between limited personal resources and ongoing work demands. From a COR perspective, the positive burnout-to-AI usage association observed in the primary model is consistent with the possibility that employees may turn to AI as an external resource when their personal resources are strained. At the same time, this pattern may not be exclusively compensatory. Greater AI usage may also reflect an avoidance-oriented response, whereby employees experiencing burnout rely more on AI to reduce the effort required for cognitively demanding tasks. Thus, both compensatory and avoidance-oriented processes may help explain the observed burnout-to-AI usage association.

The results supported a significant prospective indirect association between AI usage and job burnout through intrinsic motivation, providing qualified support for Hypothesis 2. Specifically, greater T1 AI usage predicted lower T2 intrinsic motivation, while lower intrinsic motivation was associated with higher T2 job burnout. Also, the negative prospective association between AI usage and intrinsic motivation should be interpreted as a conditional association that emerged after accounting for prior levels of intrinsic motivation and other model variables, rather than as evidence of a straightforward negative bivariate relationship. This finding suggests that AI usage may be prospectively associated with job burnout partly through lower intrinsic motivation, particularly employees’ interest in and enjoyment of the work itself. One possible explanation, consistent with self-determination theory, is that greater involvement of AI in work may alter employees’ experiences of autonomy and competence, which are important antecedents of intrinsic motivation. Although AI can improve work efficiency, when it becomes extensively involved in core tasks, employees may perceive their own competence and work value as being weakened. The sense of accomplishment that originally comes from independent thinking and creativity may also decline. Such experiences may reduce the interest, enjoyment, and sense of accomplishment that employees derive from performing the work itself, thereby contributing to lower intrinsic motivation. Although autonomy and competence were not directly assessed in the present study, these mechanisms may provide a theoretically plausible explanation for the negative prospective association between AI usage and intrinsic motivation.

Intrinsic motivation also emerged as a predictor of subsequent job burnout. Lower intrinsic motivation predicted higher subsequent job burnout. Employees with higher intrinsic motivation are generally more likely to derive interest, meaning and positive experiences from their work, and therefore maintain sustained engagement even when facing high work demands. In contrast, when intrinsic motivation decreases, employees are more likely to treat work tasks as external requirements or passive burdens. Employees need to expend more psychological resources to maintain basic performance, which makes them more vulnerable to burnout syndrome. Therefore, the weakening of intrinsic motivation could explain how AI usage indirectly raises the risk of subsequent job burnout. This finding is consistent with Rubino et al.’s (2009) argument that work stressors may influence job burnout by undermining motivation (Rubino et al., 2009). Recent research on employee collaboration with intelligent technologies also suggests that AI or robots may change employees’ perceptions of their own competence, work meaning and role value, thereby leading to negative psychological consequences (He et al., 2025). Intrinsic motivation thus represents an important psychological pathway for understanding the relationship between AI usage and job burnout. While promoting AI adoption, organizations should not only focus on efficiency gains but also attend to employees’ psychological experiences.

### 5.1. Theoretical Contribution

This study makes two main theoretical contributions.

First, this study enriches the application of COR theory to human–AI collaboration by highlighting intrinsic motivation as an important psychological pathway linking AI usage to subsequent job burnout. Although previous research has explained the negative outcomes of AI usage from perspectives like technostress, workload, or job insecurity, less attention has been paid to how AI usage may influence employees’ internal motivational resources. By adopting a two-wave cross-lagged design and accounting for prior levels of the focal variables, this study examines the prospective pathway linking AI usage, intrinsic motivation, and job burnout. By identifying a half-longitudinal indirect association between AI usage and subsequent job burnout through intrinsic motivation, this study clarifies a more specific motivational pathway linking AI usage to employee well-being. In this framework, SDT complements COR theory by providing a theoretical account of how AI usage may be associated with intrinsic motivation, whereas COR theory explains why reduced intrinsic motivation may represent a loss of motivational resources associated with subsequent job burnout. This finding suggests that the psychological implications of AI usage may be related not only to external job demands but also to changes in intrinsic motivation, reflected in employees’ interest, enjoyment, and engagement in their work.

Second, this study contributes to a more nuanced understanding of the relationship between AI usage and job burnout by examining prospective associations in both directions between AI usage and job burnout. Rather than treating AI usage only as an antecedent of employee strain, the primary analysis suggests that job burnout may also positively predict subsequent AI usage. This suggests that employees experiencing job burnout may increase their AI usage as a way of coping with depleted psychological resources and ongoing work demands. Thus, the association between burnout and AI usage may reflect a response to resource depletion, with increased AI use potentially serving either a compensatory or an avoidance-oriented function. AI usage prospectively predicted greater job burnout, while the primary model also suggested that employees experiencing greater job burnout may subsequently increase their AI usage. This pattern extends prior research that has primarily focused on the consequences of intelligent technology use by highlighting the possibility that employees’ psychological states may also be associated with their subsequent technology use.

### 5.2. Practical Contribution

The findings of this study provide several practical implications for organizations promoting AI adoption. First, from the employee perspective, the finding that AI usage positively predicts subsequent job burnout suggests that employees should use AI in a balanced and deliberate manner. Although AI can improve efficiency, employees should not hand over all core work activities to AI. When AI is involved in complex or central tasks, employees may need to spend additional effort reviewing, verifying, revising, and integrating AI outputs into their work, which may create additional cognitive and coordination demands. Therefore, employees may regard AI as a tool for improving efficiency and supporting routine or procedural tasks, rather than as a substitute for independent thinking and professional judgment. By maintaining active involvement in core work activities, employees may better preserve their intrinsic motivation and reduce the risk that AI usage becomes an additional source of burnout.

Second, from the managerial perspective, the finding of a significant prospective indirect association through intrinsic motivation suggests that managers should pay attention not only to whether employees use AI, but also to whether AI-assisted work is accompanied by changes in their intrinsic motivation. If AI implementation makes employees feel that their interest and enjoyment have been weakened, their intrinsic motivation may decline, which may further increase the risk of job burnout. Therefore, managers should consider not only the efficiency benefits associated with AI use but also its potential implications for employees’ intrinsic motivation. In practice, AI-assisted work should be designed in ways that maintain employees’ active involvement, independent judgment, and opportunities to experience competence and task ownership. Managers should also provide timely feedback and recognition so that employees can still perceive their own contribution and value in AI-assisted work.

Third, from an organizational perspective, the prospective association between AI usage and subsequent job burnout, together with the reverse association observed in the primary model, suggests that AI adoption should be supported by sufficient organizational resources. This pattern suggests that AI usage may not only contribute to later job burnout, but that burned-out employees may also increase their subsequent AI usage, potentially as a form of external support to cope with depleted energy and demanding work tasks. Without adequate organizational support, such AI use may be accompanied by additional cognitive burden and psychological depletion, potentially reinforcing the risk of job burnout. Therefore, it is essential for organizations to strengthen organizational support as an external resource that may reduce the negative psychological impact associated with AI use. Practical measures may include providing accessible and timely technical assistance, clear guidelines for appropriate AI use, reasonable workload arrangements, and channels for employees to express concerns or difficulties. Organizations can also create peer-learning mechanisms and supervisory support systems to help employees use AI more confidently and effectively. These forms of support may compensate for the psychological resources consumed associated with AI use and help employees experience AI as a supportive work resource rather than as an additional job demand.

### 5.3. Limitations and Future Directions

This study has several limitations that also suggest directions for future research. First, although the two-wave cross-lagged design improves upon cross-sectional research and allows the temporal ordering among AI usage, intrinsic motivation, and job burnout to be examined to some extent, it cannot fully establish causal relationships. The traditional CLPM does not separate stable between-person differences from within-person fluctuations. Accordingly, the cross-lagged paths in the present study should be interpreted as prospective associations rather than as evidence of within-person change processes. Moreover, the stationarity assumption underlying the half-longitudinal indirect association could not be directly tested with only two waves. The finding provides evidence of a half-longitudinal prospective indirect association, although it does not by itself establish a fully temporally ordered mediation process. Also, the one-month interval was designed to capture relatively proximal prospective associations and may not reflect the longer-term development of job burnout. Future studies using longer follow-up periods with three or more measurement waves could employ models that explicitly separate these sources of variance and examine whether these associations persist over time.

Second, the present study relied mainly on self-reported questionnaire data. Self-reports are appropriate for assessing employees’ subjective experiences, such as intrinsic motivation and job burnout, and also provide a common metric for assessing workplace AI usage across employees who may use different AI tools and platforms. The temporal separation between the two measurement waves also helps reduce some concerns associated with same-time measurement. Nevertheless, self-reported data may still be affected by social desirability, retrospective bias, and common method variance, and temporal separation cannot eliminate these concerns. Future research could therefore combine self-reported AI usage with objective indicators, such as system logs, platform usage records, or other behavioral data, to improve measurement validity and examine whether the observed associations remain consistent across measurement methods. Qualitative interviews could also complement longitudinal quantitative designs by providing deeper insight into how employees experience AI usage and the mechanisms underlying its psychological consequences.

Third, the present study focused on employees’ general level of workplace AI usage, consistent with its aim of examining the overall prospective associations of AI usage with intrinsic motivation and job burnout. This broad operationalization allowed AI usage to be examined across employees using different AI tools and applications, but it did not distinguish among specific AI technologies, functions, or usage contexts. Different forms of AI use, such as generative AI, automation, augmentation, and algorithmic management, may have different implications for employees’ motivation and well-being. Future studies could therefore examine the proposed relationships across different AI types, job types, and usage contexts, as well as potential boundary conditions such as job autonomy, leadership style, task characteristics, and AI self-efficacy. Such research could help clarify when AI usage functions primarily as a helpful work resource and when it creates additional job demands associated with burnout. Future research could also draw on SOR-based dual-path frameworks to examine both beneficial and detrimental psychological pathways linking AI usage to employee outcomes.

## 6. Conclusions

Drawing on Conservation of Resources theory, this study examined the longitudinal relationships among AI usage, intrinsic motivation, and job burnout using a two-wave cross-lagged design. The findings showed that greater workplace AI usage was prospectively associated with slightly higher subsequent job burnout and slightly lower subsequent intrinsic motivation, while lower intrinsic motivation was associated with higher subsequent job burnout over a one-month period. These findings suggest that intrinsic motivation may represent one psychological pathway linking workplace AI usage to subsequent job burnout. In addition, job burnout positively predicted subsequent AI usage in the primary CLPM, suggesting a prospective pattern consistent with the possibility of bidirectional associations between AI usage and job burnout. Dimension-level analyses further showed that this prospective association was strongest for reduced professional efficacy, weaker but still significant for cynicism, and nonsignificant for emotional exhaustion, suggesting that the prospective association was not uniform across the three burnout dimensions. Overall, this study highlights the complex psychological implications of AI usage in the workplace and suggests that AI should not be understood merely as a productivity improvement tool. Instead, organizations should pay more attention to how AI usage shapes employees’ psychological resources, work motivation, and well-being, so that AI can be implemented in ways that support rather than deplete employees.

## Figures and Tables

**Table 1:** Results of Descriptive Statistics and Correlation Analysis.

Variables	M	SD	1	2	3	4	5	6	7	8
1. T1 AI usage	10.48	4.47	—							
2. T1 IM	20.47	5.15	0.123 *	—						
3. T1 job burnout	45.83	16.21	0.286 **	−0.568 **	—					
4. T2 AI usage	11.13	4.44	0.722 **	0.049	0.309 **	—				
5. T2 IM	20.27	4.76	−0.066	0.698 **	−0.516 **	−0.076	—			
6. T2 job burnout	50.81	18.12	0.296 **	−0.573 **	0.848 **	0.362 **	−0.645 **	—		
7. Age	—	—	−0.048	0.013	0.035	−0.076	0.005	−0.038	—	
8. Income	—	—	−0.029	0.139 *	−0.046	0.033	0.109	−0.080	0.221 **	—

Note: *N* = 313. * *p* < 0.05, ** *p* < 0.01 (two-tailed). The same notation applies to all subsequent tables.

**Table 2:** Confirmatory Factor Analysis and Alternative Model Comparisons Across Two Waves.

Wave	Model	χ^2^	df	CFI	TLI	RMSEA	SRMR
T1	Five-factor	422.845	199	0.951	0.944	0.060	0.042
T1	Four-factor	1071.174	203	0.812	0.786	0.117	0.093
T2	Five-factor	400.087	199	0.959	0.952	0.057	0.036
T2	Four-factor	917.764	203	0.853	0.832	0.106	0.069

Note: The five-factor model specified AI usage, intrinsic motivation, exhaustion, cynicism, and reduced professional efficacy as distinct factors. In the four-factor model, intrinsic motivation and reduced professional efficacy were combined into a single factor.

**Table 3:** Results of Measurement Invariance Testing.

Variables	Models	χ^2^	df	CFI	RMSEA [90% CI]	SRMR	ΔRMSEA	ΔSRMR
AI usage, Job burnout, Intrinsic motivation	M1	1723.208	835	0.926	0.058 [0.054, 0.062]	0.077		
	M2	1762.272	852	0.924	0.059 [0.055, 0.062]	0.080	0.001	0.003
	M3	1842.955	874	0.919	0.060 [0.056, 0.063]	0.082	0.001	0.002

M1 = Configural invariance model; M2 = Metric invariance model; M3 = Scalar invariance model.

**Table 4:** Hypothesized Longitudinal relationships among AI usage, Intrinsic motivation, and Job burnout.

Pathway	*b*	β (95% CI)	SE	*p*
Autoregressive paths				
T1 AI usage → T2 AI usage	0.672	0.676 [0.596, 0.756]	0.041	<0.001
T1 IM → T2 IM	0.595	0.644 [0.541, 0.746]	0.052	<0.001
T1 Job burnout → T2 Job burnout	0.802	0.717 [0.644, 0.791]	0.038	<0.001
Cross-lagged paths				
T1 AI usage → T2 IM	−0.118	−0.111 [−0.204, −0.018]	0.047	0.019
T1 AI usage → T2 Job burnout	0.445	0.11 [0.04, 0.18]	0.036	0.002
T1 IM → T2 Job burnout	−0.623	−0.177 [−0.257, −0.097]	0.041	<0.001
T1 Job burnout → T2 AI usage	0.039	0.142 [0.033, 0.252]	0.056	0.011

## Data Availability

Available from the corresponding author upon reasonable request.

## Abbreviations

The following abbreviations are used in this manuscript:

AI	Artificial intelligence
IM	Intrinsic motivation
COR	Conservation of Resources
CLPM	Cross-lagged Panel Model
SDT	Self-determination theory
